# Efficient genomic DNA extraction protocol from medicinal rich *Passiflora foetida* containing high level of polysaccharide and polyphenol

**DOI:** 10.1186/2193-1801-3-457

**Published:** 2014-08-23

**Authors:** Bipin Deochand Lade, Anita Surendra Patil, Hariprassad Madhukarrao Paikrao

**Affiliations:** Department of Biotechnology, Sant Gadge Baba Amravati University, Amravati, 444602 (M.S) India

**Keywords:** DNA extraction, *Passiflora foetida*, PCR- RAPD, Phylogenetic analysis

## Abstract

In Present work, the main objective is to develop less time consuming protocol for genomic DNA isolation from leaves of *Passiflora foetida*. Optimized protocol is cost effective, as it avoided use of expensive liquid nitrogen. The important parameters of CTAB buffer composition such as Polyvinylpyrrolidone PVP40000 (without PVP, 1%, 2%, 3.5%, 4.0%, 4.5%, 5.0%), CTAB (w, 1%, 2%, 3%, 4%, 5%), water bath temperature (30°C to 70°C) and duration on water bath for half hr and one and half hr has been optimized. CTAB (2%), PVP (1%), water bath temperature (70%), duration on water bath (1 hr) has efficiently yielded DNA quality of 200-1782 μg/0.5gm from leaf, stem, root, tendril and flower. However, 168 μg - 1782 μg of DNA has been obtained from 0.5 g of leaf of *Passiflora foetida.* Polyphenol contamination has been overcome using 5M NaCl and PVP. Acetate has been used for obtaining double-stranded DNA in stabilized form. Current DNA extraction protocol takes maximum of four hours for completion, which is many time savings. RAPD-PCR reaction parameters such as DNA concentration (100ng), Primer concentration (2 μM), Dream Taq polymerase (2 U), annealing temperature (29°C) and number of cycles for amplification of DNA has been optimized. Primer fragment Akansha 7 shows high polymorphism of 7 fragments ranges from 200bp – 2500 bp. Current optimized protocol of DNA isolation is specifically for *Passiflora foetida,* which can be used for downstream molecular techniques.

## Introduction

The Genus Passiflora native to Brazil consisted of more than 600 species distributed in tropical and sub tropical part of the world, including India, Thailand, Myanmar, Indonesia, Singapore (Souza et al., 2008). In India, Passiflora is well known for unique, diversified eye catching flower, extremely used for gardening and decoration purpose. This rare feature has been attraction for extraction of genomic DNA from flower and leaves. *P. foetida* is prominent for pharmacological products that are highly acceptable in Brazil, India, America, Vietnam and European nations Ulubelen et al., ( 1982).

*Passiflora foetida* contain large secondary metabolites such as phenols, alkaloids, polyphenol and polysaccharide (Barra et al., 2012; Dhawan et al., 2002; Dhawan K et al., 2004). During DNA extraction, these compounds inhibit enzymes such as ligase, polymerase and nucleases (Barra et al. 2012) which produced negative results of restriction digestion, PCR, RAPD and cloning experiments (Howland et al., 1991; Katterman and Shattuck, 1983). Thus, in current paper such situations have been overcome by development of protocol, which isolates genomic DNA from *P. foetida*. Protocol presented here neglects the use of liquid nitrogen, which may not be available in fewer laboratories of underdeveloped and the progressing countries. All reagent and chemical used for optimization of DNA extraction has been manipulated in different combination for obtaining pure DNA from the leave sample (Suman et al., 1999; Khanuja et al., 1999; Doyle and Doyle, 1990; Doyle et al., 1987; Dellaporta et al., 1983). Same protocol has efficiently used for extraction of DNA from flower, tendrils, and stem. The research by (Crochemore et al. 2003; Fajardo et al. 1998) has concluded several species of the genus Passiflora using RAPD markers.

## Materials and methods

### Reagents and chemicals

The reagents and chemical that are used for preparation of 100 ml CTAB buffer are CTAB (Hexadecyl- trimethyl- ammonium bromide) 2.0 gm, 1 M Tris Cl (pH 8.0) 10 ml, 0.5 M EDTA (pH 8.0) 4 ml, 5.0 M NaCl 28 ml and H_2_O 40 ml, PVP 40 (polyvinyl pyrrolidine) 1 gm, adjust all to pH 5 with HCL and make volume up to 100 ml by double distilled water and other chemicals are as fallows NaOH 10 M (pH adjustment of buffer), 3.5 M sodium acetate (pH 5.3), pure cold (-20°C) isopropanol, chloroform: isoamyl alcohol (24:1 v/v), 70% ethanol, absolute ethanol, Enzyme: Dream Taq DNA polymerase (Fermentas Inc), Rnase A (Fermentas Inc), Protinase K (Fermentas Inc), Buffer: 10x Dream Taq Green buffer (Fermentas Inc), Nucleotides: dNTPs (G, A, T, C) 2 mM and RAPD primers, TAE buffer, agarose gel and ethidium bromide (Crochemore et al. 2003; Williams et al., 1990).

### Plant material

*Passiflora foetida* studied in this research were originated from India. Initially, seeds were gowned at university department garden Amravati University, Amravati (M.S) India. The young leaf samples of 40-50 mg from plants were harvested and used for optimization of the DNA.

### DNA extraction protocol

DNA extraction from *Passiflora foetida* was performed by an improved CTAB (cetyltrimethylammonium bromide detergent) method without using liquid nitrogen. DNA extraction protocol steps are as fallows.

1. Weight 0.5 gm of leaf sample of *Passiflora foetida*.
2. Grind leaf sample in mortal with the pestle in 1 ml CTAB buffer (liquid nitrogen not used).
3. Transfers to 2 ml eppendorf tubes add RNase A 20 ug/ml or (1 ul from 10 millligram/ml) mix well and incubate at 37°C for 15 min.
4. Add 10 ul proteinase k (1 mg/ml) and incubate in water bath at 65-70°C for 1 hr (Mix in between).
5. Allow to attain room temperature, add equal volume of chloroform isoamyl alcohol 24:1 i.e. 1 ml (equal volume to CTAB buffer).
6. Centrifuge at 12,000 g for 15 minutes, take supernatant carefully in fresh 2 ml eppendorf tube & discard pellet. (700 μl of the supernatant taken in the fresh 2 ml tube).
7. Add half volume of 5 M NaCl i.e. 350 μl (Mix well) keep it on ice bath for 15 min.
8. Add sodium acetate 1/10 volume of the supernatant i.e. 70 μl and add ice cold isopropyl alcohol (2/3) of the supernatant i.e. 500 μl. (Invert slowly thrice to precipitate DNA, small fiber of DNA sitting down will be observed).
9. Invert eppendroff tubes thrice and keep it -20°C for half hr.
10. Centrifuge at 11,000 g for 5 minutes, discard supernatant and take pellet.
11. Tubes containing pellet are allowed to air dry for 5–10 minutes. Invert tubes on tissue paper to complete run off supernatant.
12. Wash DNA pellet with 500 μl of 70% ethanol, centrifuge 11,000 g for 5 minutes (so that salt will dissolve, and purity of DNA will increase).
13. Discard 70% alcohol from tubes. Allow tubes containing pellet to air dry for 15 min on tissue paper in inverted position. Dissolved pellet in 100 ul NFW and stored in -20°C for further downstream procedures.

## Results and discussion

The primers sequences that are used in current study for RAPD PCR are given in Table 1. An optimization of CTAB DNA extraction buffer components such as Tris, EDTA, NaCl, PVP and CTAB (Rogers et al., 1985) are shown in Table 2. Tris interacts with the lipopolysaccharides presents on the outer membrane to denature plasma membrane and help in disruption on cell membrane. EDTA is a chelating agent it chelate Mg^++^ ions necessary for DNase activity. Thus, DNA remains protected from DNase enzyme, which requires Mg^++^ ions for its activity. DNA pellet sometime shows yellow or brown to greenish color indicates contamination of polysaccharides and polyphenol. In general, polysaccharides are very difficult to remove that interfere with a DNA isolation process (Kit and Chandran, 2010: Clark, 1997). In this experiment 5 M NaCl treatments was given to DNA in aqueous phase prior to DNA precipitation, which helps to remove polysaccharides (Kit and Chandran, 2010: Clark, 1997). Similarly, 5 M NaCl was used by (Kit and Chandran, 2010; Khanuja et al., 1999) in their protocol. Polyphenol is productively removed by using PVP, which forms a complex hydrogen bonding with polyphenol and efficiently separate it from DNA (Kit and Chandran, 2010: Doyle and Doyle, 1990). In CTAB buffer, various grades of PVP % such as without PVP, 1%, 2%, 3%, 3.5%, 4%, 4.5%, 5% as mention by (Khanuja et al., 1999) were tried. All compositions of CTAB buffer kept constant with varying PVP %. It was found that the DNA purity and concentration decrease as the PVP concentration increases. 1% PVP yields approximate 0998.7 μg from 0.5 gm of leaf sample, 2.06 and A_260/230_ = 1.99, 3.5%PVP produces 210.9-449.5 μg A_60/280_ = 1.56 ± 13 and A _260/230_ = 0.75 ± 10, 4.0% PVP yields A_260/280_ = 1.50 ± 6 and A_260/230_ = 0.67 ± 5, 4.5% PVP yields 276-282 μg A_260/280_ = 1.68 ± 3 and A _260/230_ = 0.82 ± 5, 5.0% PVP yields A _260/280_ = 1.70 and A _260/230_ = 0.81. Thus, 1% PVP has found to produce good DNA quality (Murray et al., 1980).

**Table 1:** **Representation of the primer used with their respective sequences and PCR-RAPD generated analysis of polymorphic fragments**

Primer fragment	Sequence (5’ → 3’)	References	Number of amplified fragments
Akansha 1	OPA04-AATCGGGCTG	Crochemore M. L et al., 2003	5
Akansha 2	OPB08-GTCCACACGG	Crochemore M. L et al., 2003	3
Akansha 3	OPB18-CCACAGCAGT	Crochemore M. L et al., 2003	6
Akansha 4	OPB19-ACCCCCGAAG	Crochemore M. L et al., 2003	7
Akansha 5	OPB20-GGACCCTTAC	Crochemore M. L et al., 2003	3
Akansha 6	1-CCTGGGCTTC	Aukar et al., 2003	3
Akansha 7	5-CCTGGGTTCC	Aukar et al., 2003	9
Akansha 8	53-CTCCCTGAGC	Aukar et al., 2003	3
Akansha 9	54-GTCCCAGAGC	Aukar et al., 2003	6
Akansha10	CGGGAGACCC	Chalmers et al., 1992	6

**Table 2:** **Optimization of CTAB DNA extraction buffer components for** ***Passiflora foetida***

Sr no	Parameters	Tested range	Optimum conditions	Inferences
1	CTAB	W, 1%, 2%, 3%, 4%, 5%	2%,	Disrupts cell membranes
2	PVP	W, 1%, 2%, 3%,3.5%, 4%, 4.5%, 5%	1%,	Effects DNA quality
3	Water bath temperature	30°C, 50°C, 60°C, 65°C, 70°C	70°C	Less temperature increase contamination chances
4	Duration on water bath	Half hrs, 1 hrs	1 hrs	Less duration effects DNA purity

Note: CTAB: (CTAB- catyltrimethylammonian bromide detergent) PVP:1%, W: without.

Further, grinded leaf sample in CTAB buffer was incubated on water bath and tested for temperature 55°C to 70°C. It was observed that when grinded leaf in extraction buffer kept for 65°C or less temperature, incomplete denaturation of proteins may occur which produces contamination in further extraction steps. The ground sample in CTAB buffer tested for optimization of incubation time. It was tested for minimum 25 min to 60 min. Thus 1% PVP and 70°C water bath for 1 hrs has been optimized to produce best yield of DNA. It produces 168.2 μg/ml to 1782.5 μg/ml from 0.5 g of leaf samples A _260/280_ = 1.80 ± 22 and A _260/ 230_ = 1.75 ± 20. The current protocol produces good quality and quantity of DNA when compared with another DNA isolation protocol. Krizman DNA extraction method produces 411 μg/g of leaf tissue (Abu-Romman, S. 2011).

Denaturation and removal of protein are very important to avoid its interference with DNA. Thus chloroform: isoamylalcohol (24:1) was used for denaturing proteins from DNA allowing only DNA in the supernatant after centrifugation step (Puchooa, D. 2004). At fewer moments supernatant has some greenish to yellowish color, which can be eliminated by repeating chloroform isoamylalcohole step. DNA in the supernatant was treated with half vol of 5 M NaCl (Kit and Chandran, 2010; Khanuja et al., 1999) which productively helps in removing polyphenol and polysaccharides. However, (Abu-Romman, S. 2011) has impressively used activated charcoal and PVP directly in the CTAB extraction buffer which efficiently removes polysaccharides and polyphenol. DNA is precipitated by using isopropanol and sodium acetate. In presence of water sodium acetate donates Na^++^ to DNA strand and interacts with –ve charges of the phosphate group of DNA to form complex to reduce repulsion of both strands (DNA strand –ve charge) and help to obtain DNA in an intact form. Finally, pellet of DNA washed with 70% ethanol to remove salts, a white color jelly like DNA was observed at bottom of eppendorf tube.

**Figure 1:**
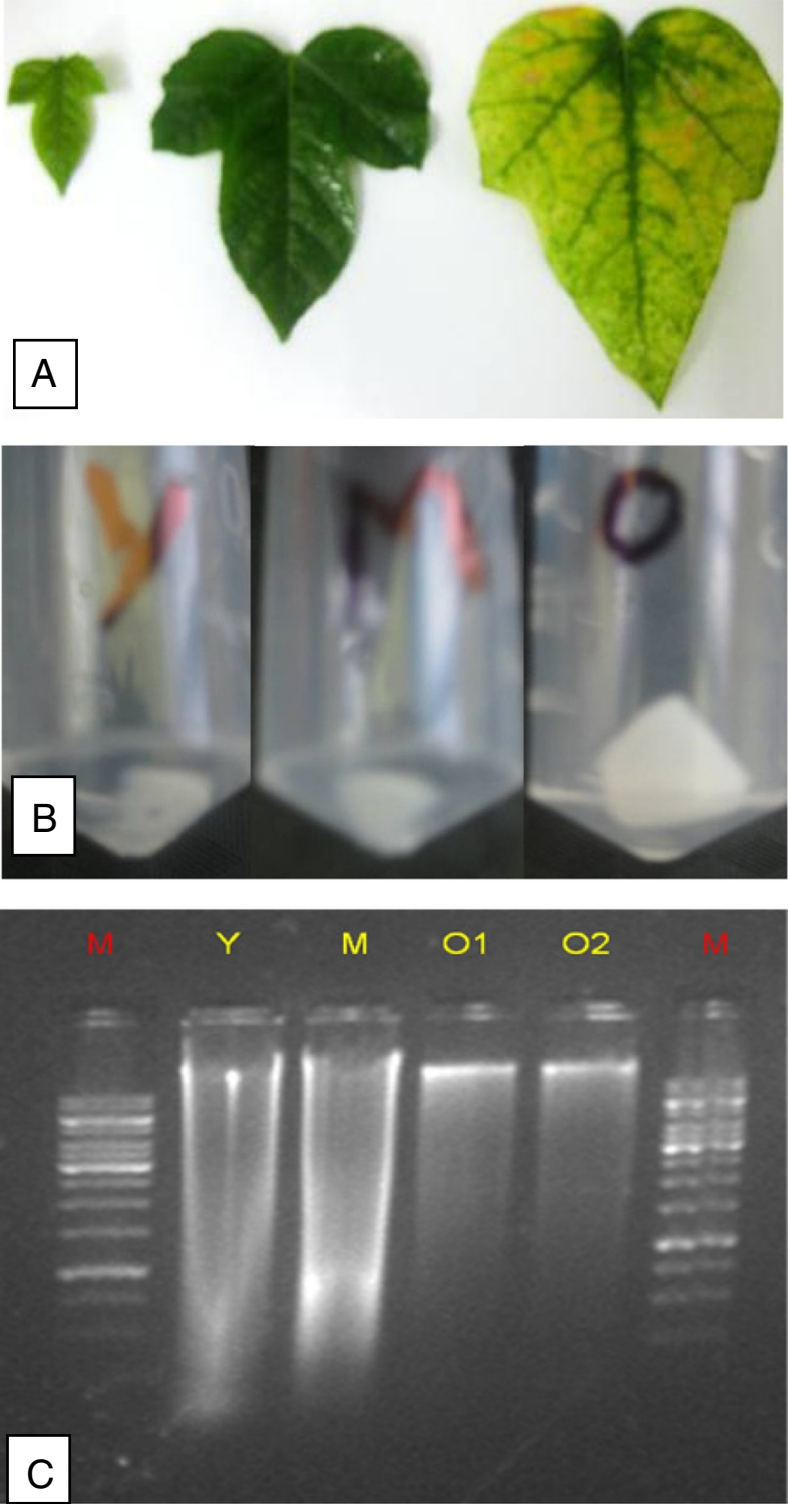
**DNA Isolated from leaf sample of** ***Passiflora foetida*** **loaded in 1.2% agarose gel, picture taken under gel documented system (Bio Rad). A**: leaf sample of young, mature, old leaf of *Passiflora foetida*. **B**: white color jelly like DNA in eppendroff tube after extraction process. **C**: DNA sample extracted from young (Y), mature (M) and old (O) leaf sample of *Passiflora foetida*.

**Figure 2:**
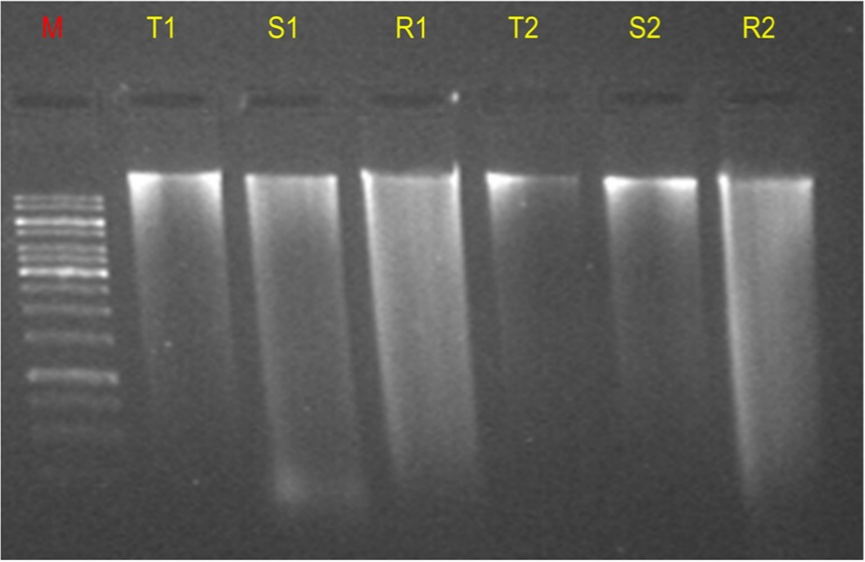
**DNA extracted from tendril, stem, and root sample of** ***Passiflora foetida.*** M: marker (1 kb), T1, T2: tendril, R1, R2: root, S1, S2: stem, 5 ul of each sample loaded in 1.2% Agarose gel, pictures taken under gel documented system (Bio Rad).

**Figure 3:**
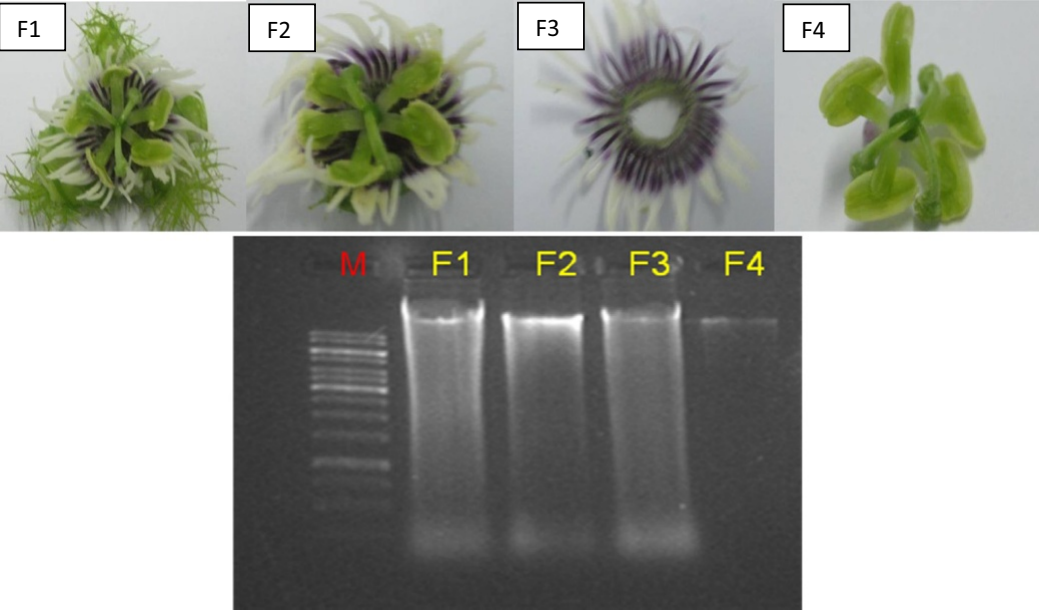
**DNA extracted from various parts of flower of** ***Passiflora foetida*** **(M: marker 1 kb. F1: whole flower, F2: without sepals, F3: without reproductive system, F4: reproductive system only).** 5 ul of each sample loaded in 1.2% Agarose gel, picture taken under gel documented system (Bio Rad).

Thus, our optimized protocol for DNA extraction from 0.5 g of leaf sample yield's DNA of 168 μg-1782 μg/ml. Figure 1 shows isolated DNA from leaf sample (young, mature, old) of *Passiflora foetida* that were loaded in 1% agarose gel and picture taken with gel documentation system. Same protocol has been used successfully for isolation of DNA from stem, root, flower and tendril. Figure 2 shows DNA in agarose gel electrophoresis from stem, tendril, root and Figure 3 shows isolated DNA from various flower parts (Rogers et al., 1985). The DNA from Figure 3: F1: (whole flower), 3: F2 (without sepals), 3: F3 (without reproductive system) shows slight degradation, however, DNA from 3: F4 (reproductive system) is pure and intact without degradation.

Annealing temperature = 4 (G + C) + 2 (A + T)

**Figure 4:**
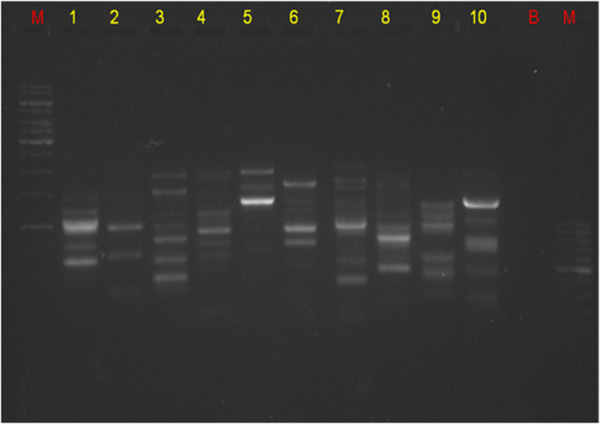
**Optimized Akansha primer 1,2,3,4,5,6,7,8,9,10, M: 1 kb ladder (right) and 100 bp (left) ladder, B: blank, PCR- RAPD Analysis - of** ***Passiflora foetida.*** Loaded in 1.2% agarose gel, picture taken under gel documented system (Bio Rad).

At last, PCR cycles affect the amplification of DNA and 30 PCR cycles produces good DNA amplification. Optimization of the RAPD-PCR reaction parameters for amplification of genomic DNA of *Passiflora foetida* are displayed in Table 3*.* The PCR reaction was performed in triplicate for reproducibility of amplified 51 fragments, which ranges from (120 bp -2500 bp). Figure 4 shows optimized Akansha RAPD-PCR primers 1-10. The primer no 7 shows highest biased power and 2, 5, 6, 8 show low discriminatory power Williams et al., ( 1990).

**Table 3:** **Optimization of the RAPD-PCR reaction parameters for amplification of genomic DNA of** ***Passiflora foetida***

Sr no	PCR parameters	Tested range	Optimum conditions	Inferences
1	DNA concentration (ng)	50, 75, 100, 150, 200	100 ng	Less amplification with lower concentration and smear formation at higher concentration
2	Primer concentration (μM)	1, 1.5, 2, 2.5, 3, 3.5, 4, 4.5, 5.	2 μM	Minimum amount produces sufficient amplification
3	Dream Taq polymerase (units)	2 U, 5 U	2 U	Sufficient for proper amplification.
4	Annealing temperature (°C)	25, 27, 29, 30, 35, 40,44	29°C	Lower annealing temperatures show proper annealing and amplification
5	No of cycles	25, 30, 35, 40, 45	30	Higher/lower cycles (from optimum) effects the amplification

## Conclusion

Thus present protocol denied the use of liquid nitrogen is inexpensive, easy, and less time-consuming effectively yields genomic DNA from the fresh and dry sample. The high level of secondary metabolites from this plant makes extraction difficult. However, optimized protocol flourishing isolates genomic DNA that holds promise for further high-throughput molecular techniques, including AFLP (Segura et al. 2002), RFLP (Botstein et al. 1980), PCR extension and RAPD (Crochemore et al., 2003). Genomic DNA from leaf, tendril, stem, root and flower has been extracted successfully. RAPD analysis can be done prosperously for improvement of fruiting varieties of Passiflora species.
